# Rapid response to climate change in a marginal sea

**DOI:** 10.1038/s41598-017-04455-5

**Published:** 2017-06-22

**Authors:** K. Schroeder, J. Chiggiato, S. A. Josey, M. Borghini, S. Aracri, S. Sparnocchia

**Affiliations:** 1 CNR-ISMAR, Venice, Trieste, La Spezia Italy; 2 National Oceanography Centre, University of Southampton Waterfront Campus, European Way, Southampton, SO14 3ZH UK

## Abstract

The Mediterranean Sea is a mid-latitude marginal sea, particularly responsive to climate change as reported by recent studies. The Sicily Channel is a choke point separating the sea in two main basins, the Eastern Mediterranean Sea and the Western Mediterranean Sea. Here, we report and analyse a long-term record (1993–2016) of the thermohaline properties of the Intermediate Water that crosses the Sicily Channel, showing increasing temperature and salinity trends much stronger than those observed at intermediate depths in the global ocean. We investigate the causes of the observed trends and in particular determine the role of a changing climate over the Eastern Mediterranean, where the Intermediate Water is formed. The long-term Sicily record reveals how fast the response to climate change can be in a marginal sea like the Mediterranean Sea compared to the global ocean, and demonstrates the essential role of long time series in the ocean.

## Introduction

**Figure 1:**
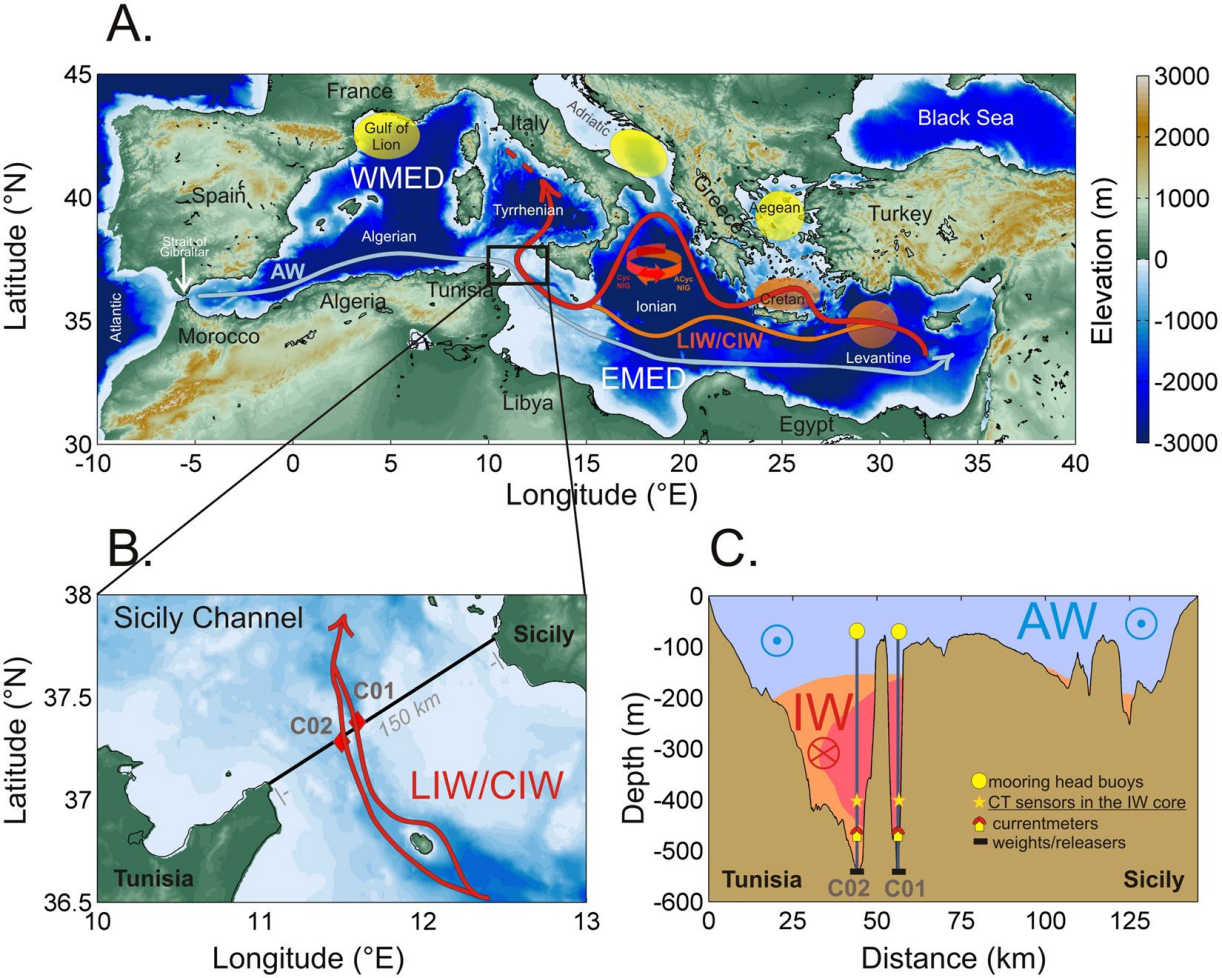
(**A**) The Mediterranean Sea where deep (yellowish ellipses) and intermediate (reddish ellipses) water formation sites are highlighted as well as the circulation schemes for the Atlantic Water (AW, light blue arrow) and the Levantine/Cretan Intermediate Waters (LIW/CIW, red arrow), the black rectangle indicates the monitored area; (**B**) zoom on the Sicily Channel where the positions of the two moorings (C01 and C02) are shown (red diamonds), that allow both branches of the bifurcated LIW/CIW flow (red arrow) to be intercepted; (**C**) vertical schematic section of the 150 km-long transect between Tunisia and Sicily (black line in (**B**)), showing the two-layer system of water masses flowing in opposite directions (AW in light blue flowing eastward; IW in orange flowing westward, where the darker orange indicates higher salinity, the salinity maximum identifying the core of the IW) and the positions of instruments along the C01 and C02 lines located in two parallel deep trenches. The data discussed in the paper come from the conductivity-temperature sensors at about 400 m depth, located in the intermediate water (IW) (the yellow stars). Maps were generated by using MATLAB 7.1 http://uk.mathworks.com/products/matlab.

Because of their small volume to surface area ratio, marginal seas tend to respond to global warming and to changes in freshwater inputs (via evaporation-precipitation-river runoff, E-P-R) much more strongly than the open ocean. The Mediterranean Sea (Fig. 1A) is an outstanding example for this: it has much shorter turnover timescales (1/10th) than the global ocean^1^ and it is an evaporation-dominated region for which a further decrease in precipitation is projected in the twenty-first century as a result of anthropogenic climate change^2, 3^. In addition, the second half of the twentieth century has seen a high rate of dam constructions for Mediterranean rivers, the building of the Aswan Dam in 1964 being the most impactful intervention, which greatly reduced (by about 50%, according to ref. 4) the freshwater input to the sea.

The Mediterranean is connected to the global ocean by the Strait of Gibraltar (sill depth ≈300 m) and is formed by several regional sub-basins, separated by a number of channels^5^. The most important one, after Gibraltar, is the Sicily Channel (SC, sill depth ≈500 m, Fig. 1B and C), a key choke point, where all water masses exchanged between Eastern Mediterranean (EMED) and Western Mediterranean (WMED) can be intercepted. This makes it possible to observe the variability of heat and salt carried by the most ubiquitous Mediterranean water mass, the Intermediate Water (IW), which forms in the EMED and flows towards the WMED, eventually exiting towards the Atlantic Ocean.

The inflow of fresh surface Atlantic Water (AW), the evaporation excess over the basin and strong heat losses during winter in specific areas, drive an antiestuarine circulation, i.e. a circulation characterized by the inflow of low-salinity surface water over a deeper outflowing, dense, high-salinity water layer (the IW). As the AW spreads through the basin it is modified: in the EMED it is transformed into salty and warm IW, called Levantine Intermediate Water (LIW) or Cretan Intermediate Water (CIW), depending on the specific formation location (Levantine or Cretan Sea, respectively, Fig. 1A). The formation of these water masses is driven by wind-induced strong evaporation and heat loss during winter, both processes increasing the density of the surface layer until it sinks via convection to an intermediate depth (about 150–300 m)^6^. The LIW/CIW layer is identifiable in the whole Mediterranean Sea by a subsurface S maximum. While flowing back towards the WMED, crossing the SC, it tends to gradually lose its characteristics, due to dilution with adjacent water masses, becoming thus less salty and less warm (and less oxygenated due to respiration processes). After the SC the LIW/CIW is topographically forced to steer north-eastward and enter directly the Tyrrhenian Sea, and eventually reaches the northern WMED. The role of LIW/CIW is crucial in determining the amount and characteristics of the deep waters^7^, that are formed in and ventilate both the EMED and the WMED (see locations in Fig. 1A). The heat and salt content of the IW preconditions the formation process and in the long-term determines the thermohaline characteristics of the deep waters. Finally, the LIW/CIW forms the bulk of the Mediterranean Outflowing Water (MOW), which exits through Gibraltar and settles down to about 1000 m in the Atlantic due to its high density. Multidecadal salinity increases of the order of 0.0013 yr^−1^ have been observed in the Atlantic Ocean at these depths between 30°N and 40°N, corresponding to the heart of the MOW influence, and have been ascribed by refs 8 and 9 to the salinity increases occurring in the Mediterranean, which are shown in the following section.

## Results

**Figure 2:**
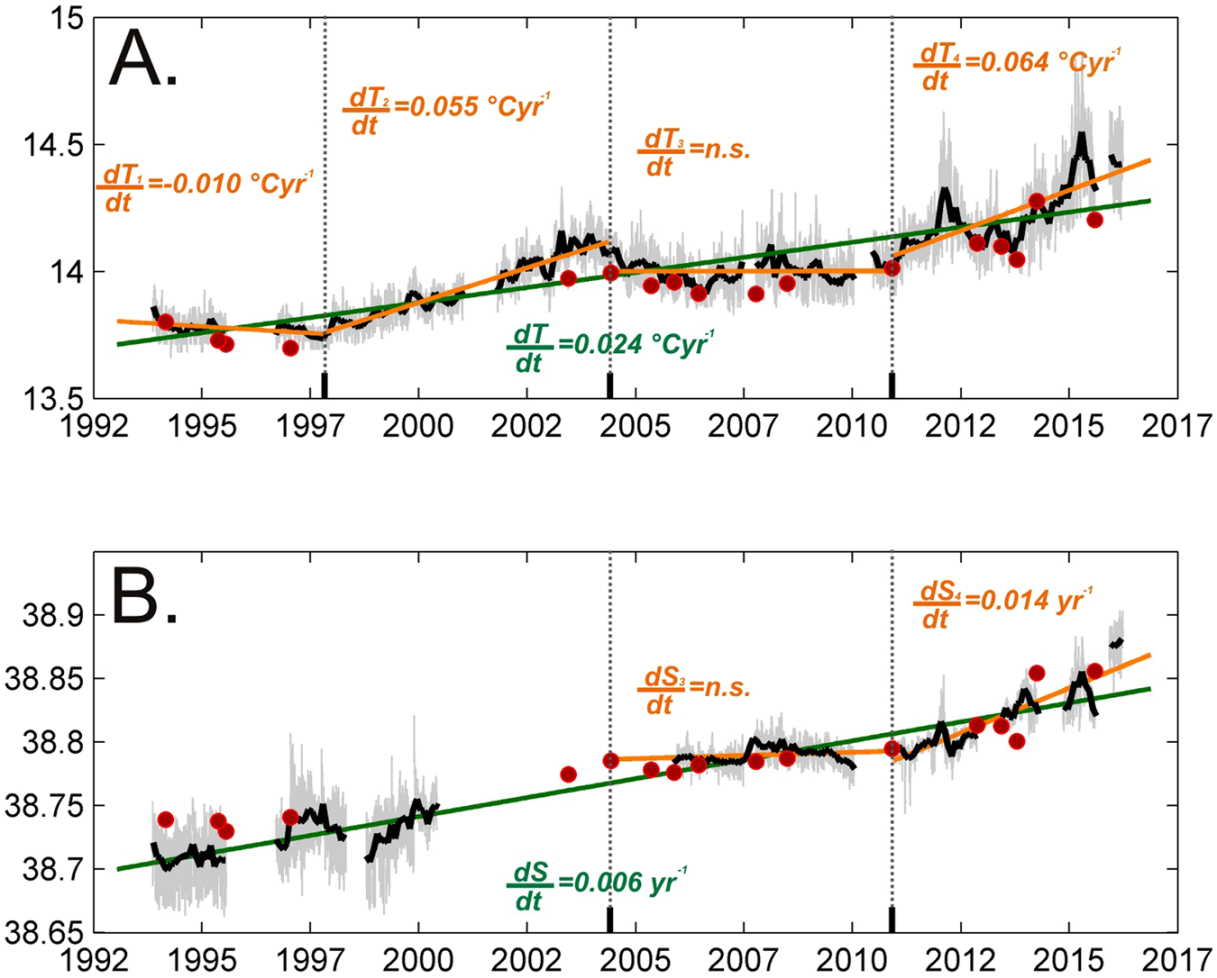
Time series (3-hourly in light grey) of (**A**) temperature and (**B**) practical salinity at 400 m depth (mooring C02). Red dots indicate CTD measurements from ship during servicing. The monthly mean time-series is shown in black. The green line represents the long-term trend line while short-term trends found for different periods detected by means of the change point analysis are shown in orange. For each trend line the trend value is given, if significant, in the same colour as the corresponding line. The vertical dashed lines indicate change points.

The time series of temperature (T) and salinity (S) at 400 m depth show notable interannual variations and long-term trends, with both tracers co-varying (Fig. 2). Since the beginning of the time series (22 years ago), the T of LIW/CIW has increased by about 0.53 °C and its S by about 0.13, thus with mean trends of 0.024 °C yr^−1^ and 0.006 yr^−1^, respectively. It is evident in Fig. 2 that the thermohaline trends are subject to changes, slowdowns and accelerations throughout the 22 years, related to a number of forcings, some of which have been explored in previous studies [e.g refs 10 and 11.] and others that will be discussed here for the first time.

A change point analysis^12^ identifies an initial period, up to 1997, when T decreased (−0.01 °C yr^−1^). This period was under the influence of the Eastern Mediterranean Transient (EMT)^13^, during which an uplifting of old Eastern Mediterranean Deep Water allowed its upper part to cross the SC, with a consequent T reduction^14, 15^. The S signal was less intense, visible mainly in 1991–1993, prior to the start of the mooring record^14^. The successively observed S increase, up to mid-2000, is consistent with previous studies^14, 16, 17^.

Among others^18^, have related the internal EMED salt distribution to a periodic reversal of the Northern Ionian Gyre (NIG, see Fig. 1A)^19^. An anticyclonic phase (that occurred during 1993–1997 and 2006–2011)^19^ inhibits the AW advection to the southern EMED and favours its deflection towards the Adriatic. Conversely a cyclonic phase (that occurred during 1998–2005)^19^ leads to a stronger dilution in the southern EMED. Consequently, an anticyclonic NIG favours the production of salty and warm LIW/CIW, and a cyclonic NIG does not. Other authors^20, 21^ prefer to speak about a “thermohaline pump”: a quasi-decadal oscillation between competing dense water formation regions that forces the AW to be mainly advected to the Levantine or the Adriatic. The main difference between these two postulated mechanisms consists in that *cause* and *effect* are inverted. What matters here is that they act at the same temporal scales and have the same effect on LIW/CIW. This effect seems to be detected in the SC choke point with a temporal lag of about 4–5 years, although this interpretation remains unaddressed yet and deserves further research.

During fall 1997 – summer 2004 an increase of the T trend occurred (0.055 °C yr^−1^). The S time series does not cover most of this period, but the approximate raise is of the order of 0.05 in 7 years. This period comes immediately after a period of anticyclonic NIG and the augmented trends in the SC are likely to be a response to a warmer and saltier IW production during this phase. In this context, we note that^22^ reports two major peaks in LIW T and S in the Levantine (1992 and 2008), and relate them to a contemporary anticyclonic regime of NIG.

The period summer 2004 – winter 2010/2011 sees an abrupt end of the increasing trends in the SC. This period follows a long cyclonic phase of NIG, which is the most plausible candidate to explain this behaviour. Since then (winter 2010/11 – spring 2016) the thermohaline trends of LIW/CIW have undergone a dramatic increase, each year reaching higher peaks (during the 22 years the maximum values of T and S were reached in spring 2015, with T_max_ = 14.86 °C, and spring 2016, with S_max_ = 38.9, respectively). The T and S trends are now about 2.5 times stronger than the overall trends: 0.064 °C yr^−1^ and 0.014 yr^−1^, respectively. The NIG in 2006–2011 was indeed anticyclonic again, which might have favoured the production of saltier and warmer IW.

Previous studies reported much lower trends for the IW in the Mediterranean Sea, e.g ref. 23. reported mean trends for 1943–2000 of 0.004 °C yr^−1^ and 0.002 yr^−1^, respectively for T and S, in the WMED. Using only the CTD data collected in the SC (red dots in Fig. 2), we found slightly (but not significantly) lower trends (0.02  °C yr^−1^, for T, and 0.005 yr^−1^, for S), which are still higher that those calculated by ref. 23. These differences are potentially related to at least three aspects: (i) the trend increased recently, during 2010–2016 (it has more than doubled the longer term trend values), (ii) the SC record provides an “integral“ assessment of what happens in the EMED, while most previous studies were focusing on different regions (mainly of the WMED), where the signal carried by the IW could have been diluted by spreading along different pathways, and (iii) assessing long term trends based on continuous eulerian measurements gives different results to assessments based on sparse, in time and space, CTD casts. Indeed, only the very recent study by ref. 22, analysing CTD data in the Levantine basin from 1979 to 2014, reports trends for LIW T and S that are comparable to those reported here, i.e. 0.03 °C yr^−1^ and 0.005 yr^−1^.

Furthermore, the trends observed in the SC are at least one order of magnitude greater than those reported for the global ocean intermediate layer (ref. 24 dT/dt = 0.003 °C yr^−1^, 0–700 m, global, 1955–2010; ref. 25 dT/dt = 0.0006–0.0013 °C yr^−1^, 100–300 m, global, 2004–2013; ref. 26 dT/dt = 0.005 °C yr^−1^, 0–500 m, global, 2006–2013; ref. 27 dS/dt = 0.0002–0.0006 yr^−1^, 200–700 m, Atlantic Ocean).

Overall, the 22-year-long record shows a superposition of different time scales of variability: (i) high frequency episodes (daily to annual, not discussed), (ii) prolonged periods (6–7 years) of decreased/increased trends (ascribed to internal variability, exceptionally intense wintertime atmospheric forcings, NIG reversals, EMT), and (iii) longer term increasing trends (>10 years). Now that the time series is growing longer, revealing the ceaseless nature of T and S increases, the effect of global warming (and in particular of “enhanced regional warming in a hot temperature extreme” like the Mediterranean region, see ref. 28) needs to be taken into account, as will be discussed in the following section.

## Discussion

In order to explain long-term changes in the properties of a water mass transformation product, it is necessary to focus on the “hydrographic” preconditioning^29, 30^: i.e. the (increased) heat content and salinity of the water masses that contribute to the water mass formation. The (atmospheric and oceanographic) conditions within the formation areas during winter are critical in determining the properties of the water formed in that particular year. In fact, the process of water mass formations is localized in time and space and the newly formed water is rapidly advected out of the area^31^. This is why the long-term forcing that causes the observed overall trends in the LIW/CIW should not be sought in their formation regions, but rather in those regions that contribute to set the “hydrographic” preconditioning, i.e. “upstream” of the formation area. A warmer and drier regional climate in the EMED, as well as a reduced freshwater input from rivers^10, 11^, favours the formation of warmer and saltier IW, given that LIW/CIW is formed by transformation (densification due to evaporation and cooling) and sinking of Levantine Surface Water (LSW), either in the Levantine^6^ or in the Cretan Sea^32^. LSW originates from the modification of AW along its path through the Ionian and the Levantine, where it becomes particularly warm and salty. Hence the climatic conditions along this path are likely to be crucial in eventually determine the heat content and the salinity of Mediterranean IW. In this regard, it is worth noting that^33^ reports a recent drought in the Levant (since 1998) which is the driest in the past 500 years. The small size of the Mediterranean and its being enclosed between continents renders this “miniature ocean”^1^ particularly sensitive to changes in external forcings and climate. Indeed, recent studies indicate the Mediterranean to be one of the most responsive areas to climate change^34^.

Long-term analyses of fixed moorings, ARGO floats and ship-based CTD data^21, 22, 35, 36^ suggested that there are important intermediate T and S peaks in both areas (Levantine, Cretan). In particular^36^, analyzed the long-term variability of the water column properties in the Cretan Sea (1986–2014) based on historical CTDs and found that after S peaks in 1993 and 1999, between 2008 and 2011 the S of CIW has increased by 0.2–0.25. According to refs 21 and 35 this is a manifestation of the salinity preconditioning of the Cretan Sea (due to mechanisms such as NIG reversals or the thermohaline pump mentioned before), i.e. a recurrent phenomenon. But what is not considered in these studies is that there is also a tendency of the peaks (in the Levantine and in the Cretan Sea, as appears from the datasets published by refs 21, 35 and 36) to reach greater values, whose causes cannot be found in the local hydrographic and atmospheric conditions. Instead they occur “upstream” reflecting the increasingly strong water mass transformation that AW and LSW experience while on their path towards the formation regions of LIW/CIW. The same tendency is observed in the SC where from our time series we estimated a trend in the T and S peaks by fitting a linear regression model to the annual maximum T and S values: in both cases a significant (p-values ≪ 0.01) increasing trend of 0.036 °C yr^−1^ and 0.0046 yr^−1^, for T peaks (R^2^ = 0.84) and S peaks (R^2^ = 0.75) respectively, was found.

**Figure 3:**
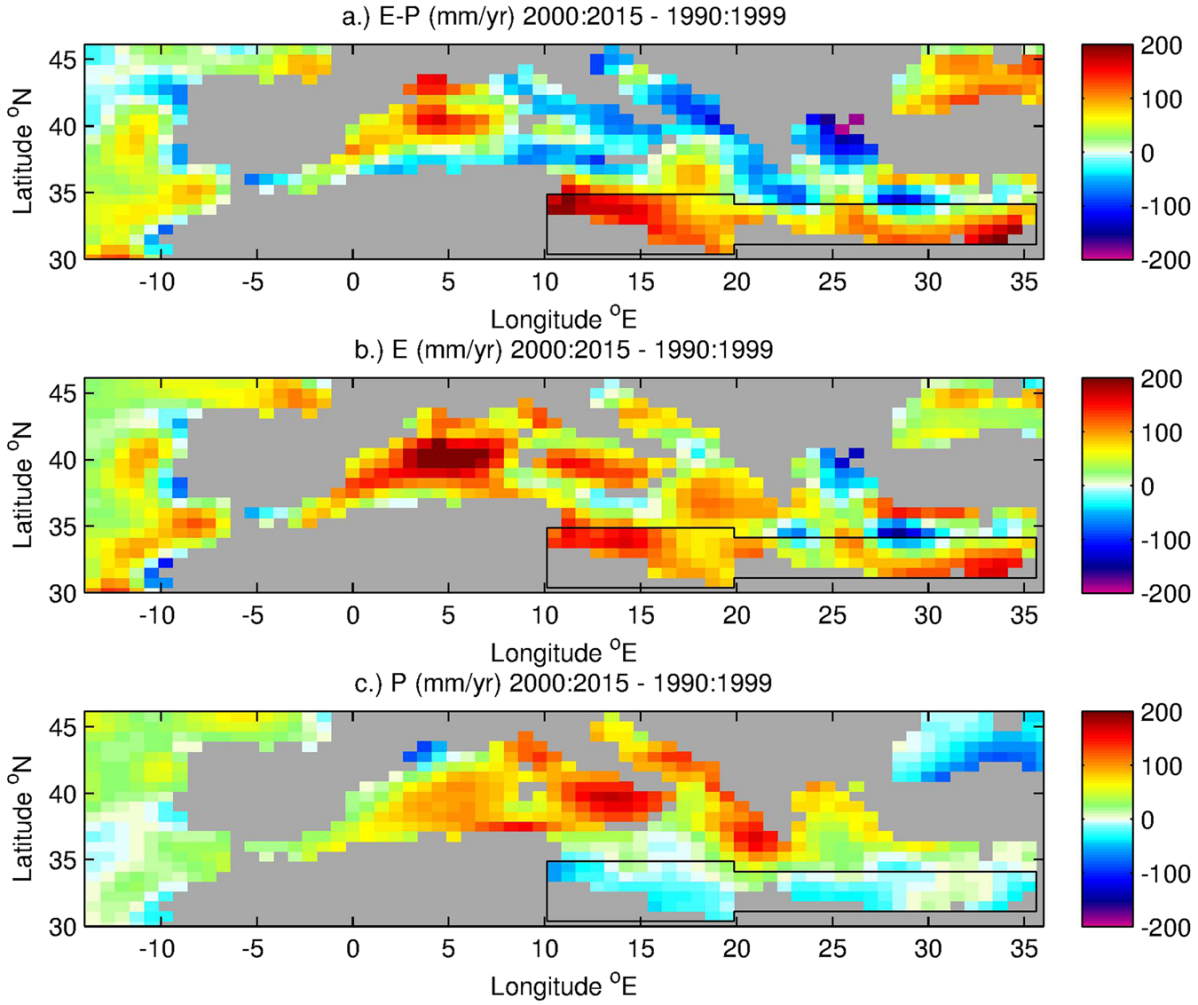
Difference of ERA-Interim annual mean surface freshwater flux and its components averaged over 2000–2015 from 1990–1999. (**a**) Evaporation – Precipitation (mm yr^−1^), (**b**) Evaporation (mm yr^−1^) and (**c**) Precipitation (mm yr^−1^). The black outline shows the south-eastern Mediterranean box used to determine the time series shown in Fig. 4.

**Figure 4:**
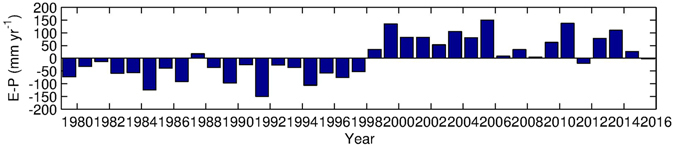
ERA-Interim annual mean surface freshwater flux (E-P in mm yr^−1^) anomaly (relative to 1979–2013) for the south-eastern Mediterranean box shown in Fig. 3.

So, in contrast to other studies that explored (winter) atmospheric conditions over the Cretan or the Levantine, we investigate whether the transformation of AW into a saltier and warmer LSW in recent decades is likely due to changes in the net evaporation (E-P) over the EMED, and in particular in its southern part. Also continental freshwater input (R) reduction has been considered as a plausible candidate to induce salinification of the Mediterranean waters [e.g ref. 10]: the construction of major dams during the 1950s was able to produce significant S increases from surface to bottom in the EMED. The reduction of R may also play a role in the formation of warmer and saltier water masses [e.g refs 10, 11, 37 and 38, such studies indicate that environmental driving forces should also be considered alongside climatic factors. However, the impacts of dam construction are no longer felt after about 40 years^37^. Consequently, damming in the 1950s is not a major influence in the period that we are considering and the cause of the recent changes must be found elsewhere than river runoff. Thus, here we focus on the E-P components of the freshwater budget. The change in the Mediterranean surface E-P field from ERA-Interim, together with E and P component changes, are shown in Fig. 3. The maps show the difference of 2000–2015 from 1990–1999 (these periods are chosen as the time series in Fig. 2B reveals a strong increase in salinity values between the 1990s and 2000s). In particular, the E-P increase between the two periods in the south-eastern Mediterranean (Fig. 3a) is mostly due to an E increase (Fig. 3b) that is reinforced to some extent by a slight reduction in P (Fig. 3c). The figure shows a change in E-P in the whole southern part of the EMED, that coincides with the eastward pathway of the surface water, a pattern that is consistent with increasing warming and salinification of this layer and a consequent T and S increase in the IW in the SC. Further support for this conclusion is provided by the E-P anomaly time series (Fig. 4) for a box that covers the area of the AW pathway in the southern EMED (black outline in Fig. 3, note the time period 1979–2015 has been selected as it covers all available data from the ERA-Interim reanalysis employed for our investigation). A noticeable switch in the sign of the anomalies is evident in 1998 (coherent with the findings by ref. 33) and is maintained through to 2014 revealing a prolonged intensification of net evaporation in this region.

The increasing import of salt and heat from the EMED to the WMED, via the IW, will have (and already had) significant consequences. Once advected to the dense water formation region in the Gulf of Lion, more salt and heat in the IW will further enhance the tendency of this site to produce warmer and saltier deep waters^30^. This process started in 2005 with the advent of the Western Mediterranean Transition (WMT^1^) and will ultimately have an impact on the MOW^8, 9^. The strong changes in the Mediterranean Sea stand out when compared with globally averaged trends, indicating the potential for regional amplification of climate signals. Note that it is also possible that regional modes of climate variability, principally the North Atlantic Oscillation and East Atlantic Pattern, play some role through their impact on regional evaporation and precipitation. However, for the period from the early 1990s to mid 2010s, the NAO and EAP show strong interannual variability in their index values rather than a prolonged trend^39^, thus unforced climate variability arising from these modes is not expected to be a significant factor in our analysis.

The ongoing climate change has certainly increased the scientific interest in time series analyses and their importance is increasingly recognized even at the political level. This time series contributes to explicitly show how long oceanographic time series are fundamental for climate studies. Nonetheless, due to their high maintenance costs and the difficulty in establishing and maintaining an observing system they are still widely lacking, both at the global scale and in marginal seas. It is important to stress that an understanding of physical (as well as chemical and biological) processes in the oceans requires regular and long-term observations, that enable us to separate real long-term trends in environmental drivers from the natural variability of the system.

## Methods

The SC site has been monitored for thermohaline properties and water mass exchanges since 1993 by two moorings located within parallel trenches in the 150 km-long transect between Tunisia and Sicily (Fig. 1C). They form two of the longest Mediterranean time series of thermohaline properties and are part of the HYDROCHANGES network (Mediterranean Science Commission, CIESM)^40^. Due to the Coriolis effect, the IW core at the sill that funnel its westward flow is squeezed to the right, in both trenches (Fig. 1B), with the 400 m deep record in the north-eastern mooring (called C01) showing slightly warmer (by about 0.04 °C) and saltier (by about 0.01) values than the south-western one (called C02). As the temperature (T) and salinity (S) time series at both sites are very similar only the C02 time series is shown here. As an additional point of reference, Fig. 2 shows also the values retrieved by CTD casts performed at the mooring location during each servicing cruise. The nominal depth of the instruments is 400 m, which is however subject to slight variations between servicing of the mooring, of the order of few tens of meters.

From 1993 to November 2002 temperature and conductivity have been recorded by means of Aanderaa RCM7 current meters, with an accuracy of 0.05 °C and 0.05 for T and S, respectively. Since November 2002, SBE37 probes have been used, which further improved the quality of the measurements, yielding 0.002 °C and 0.001 for T and S, respectively. Both types of instruments (RCM and SBE) have been regularly calibrated (at least on an annual basis). The order of magnitude of the changes observed over the 22 years is such that the difference in accuracy does not affect the computation of trends. These trends have been computed using linear regression on monthly means and t-tested for significance (α = 0.01) using MATLAB®. In order to detect short-term trends, a change-point analysis^12^ has been run on monthly means of T using MATLAB®, with gaps filled with linear interpolation. After visual inspection and following the posterior distribution of selecting a given number of change points, the maximum number (k_max_ in ref. 12) was set to 3, while the temporal distance between change points (d_min_ in ref. 12) was set to 1 year. Results are rather insensitive to other choices of d_min_, e.g. up to 5 years. A d_min_ longer than 5 years instead would not be consistent with the selected k_max_ considering the length of the time-series. Due to substantial gaps in the S time-series, the change points detected based on T time-series have been assumed to be the same for the S time-series.

ERA-Interim evaporation and precipitation fields have been obtained from the European Centre for Medium Range Weather Forecasting (http://www.ecmwf.int/en/research/climate-reanalysis/era-interim). Anomalies have been calculated with respect to the period 1979–2013.
